# Supplementary material for: The interactome of intact mitochondria by cross-linking mass spectrometry provides evidence for coexisting respiratory supercomplexes
Source: Mol Cell Proteomics. 2017 Dec 8;17(2):216–32. doi: 10.1074/mcp.RA117.000470 (PMC5795388; doi:10.1074/mcp.RA117.000470)
Supplement: Supplemental Data [file supp_RA117.000470_133922_0_supp_23978_fzffwf.zip › spectra_annotation/mito_DR_spectra_annotation/141-1-15-1-6-1.pdf]

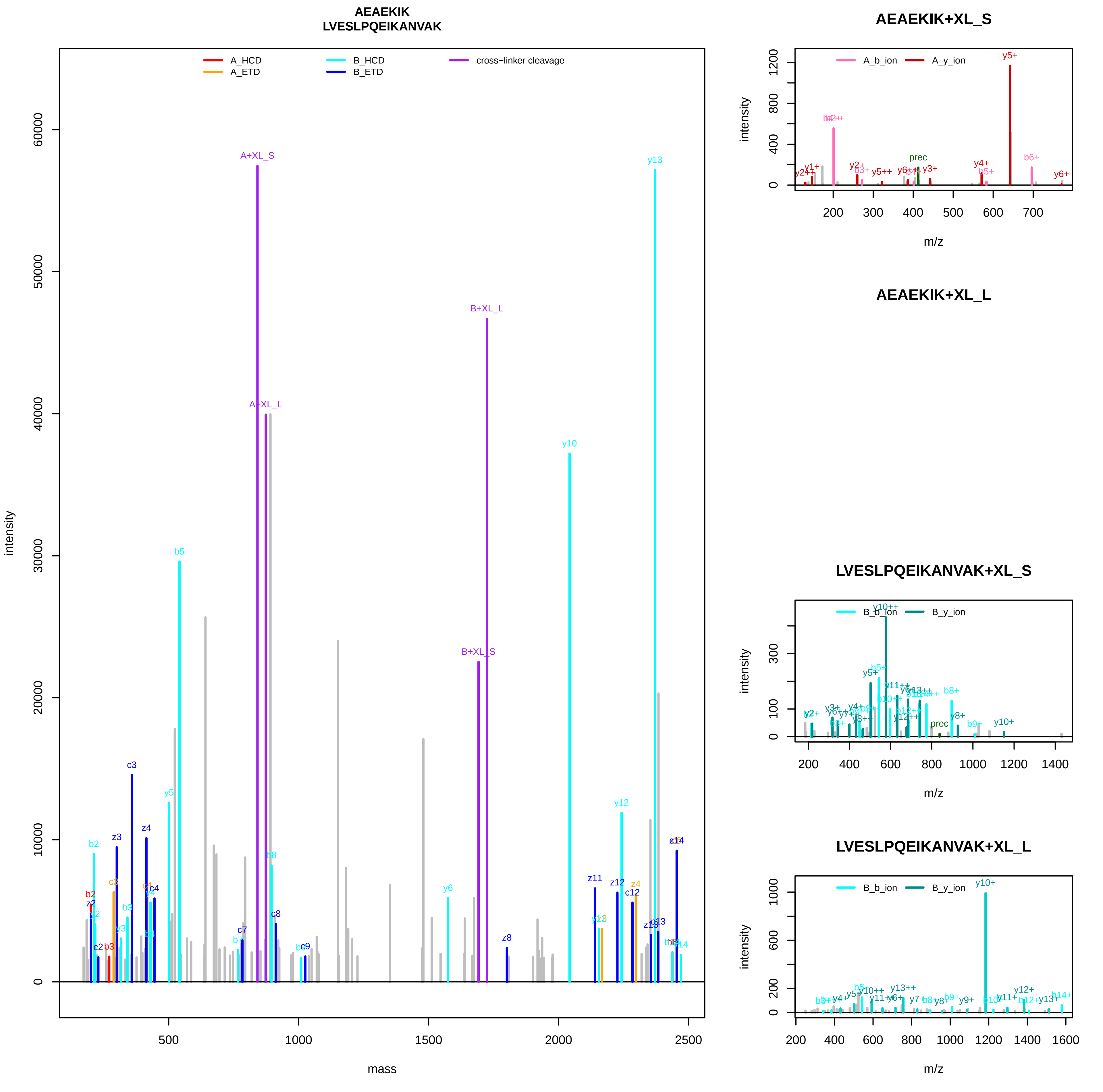
AEAEKIK
LVESLPQEIKANVAK
A_HCD
A_ETD
B_HCD
B_ETD
cross−linker cleavage
A+XL_S
A+XL_L
B+XL_L
B+XL_S
y13
y10
b5
y5
c3
z3
z4
b2
y12
z11
z12
c12
z4
c8
b8
y6
z8
c9
c7
b7
z13
c13
z14
y14
intensity
mass
0
10000
20000
30000
40000
50000
60000
500
1000
1500
2000
2500
AEAEKIK+XL_S
A_b_ion
A_y_ion
y5+
b6+
y4+
b5+
y3+
prec
y6++
y5++
y2+
b3+
b2++
y1+
y2++
y6+
m/z
AEAEKIK+XL_L
LVESLPQEIKANVAK+XL_S
B_b_ion
B_y_ion
y10++
b5+
y5+
y11++
b8+
y8+
b9+
y10+
prec
LVESLPQEIKANVAK+XL_L
y10+
y13++
b5+
y12+
b14+
y13+
y11+
y9+
y7+
y4+
y5+
